# Medication Management Using Technology-Based Interventions in Older People at Home Care Settings: Protocol for a Scoping Review

**DOI:** 10.2196/75200

**Published:** 2025-10-03

**Authors:** Shiji Thomas, Beth Fylan, Jonathan Silcock, Md Shafiqur Rahman Jabin

**Affiliations:** 1 School of Health and Society, University of Salford, Manchester, United Kingdom; 2 Faculty of Health and Social Care, University of Bradford, Bradford, United Kingdom; 3 Faculty of Life Sciences, University of Bradford, Bradford, United Kingdom; 4 National Institute for Health and Care Research Yorkshire and Humber Patient Safety Research Collaboration, Bradford, United Kingdom; 5 Department of Medicine and Optometry, Linnaeus University, Pedalstråket 11, Kalmar, 392 31, Sweden, 46 7915 673 612

**Keywords:** complex social care, effectiveness, medication management, patient safety, digital health intervention, personalized care, decision support system

## Abstract

**Background:**

Older adults with complex care needs and polypharmacy are at risk of experiencing medication-related problems such as administration errors and adverse drug events. These problems lead to increased use of emergency care and hospital admissions. Technology-based interventions have been introduced to address some of these problems. This review aims to establish key concepts and address the gaps in the evidence base to improve the use of technology-based interventions and reduce medication management-associated problems in home care settings.

**Objective:**

This scoping review aims to understand the extent and type of evidence regarding the management of medications for older people in home care settings using technology-based interventions.

**Methods:**

The review will follow the Joanna Briggs Institute (JBI) methodology. The published studies will be searched through MEDLINE, CINAHL, Scopus, Web of Science, Embase, IEEE Xplore, and ACM digital library, and the unpublished studies through the EBSCO Open Dissertations database. Studies published in all languages will be considered. A broad scope of evidence, including quantitative, qualitative, text, and opinion studies, will be considered. This review will include studies of older adults (aged 60 years or older) living in home care settings and receiving formal or informal social care support for medication management. Studies that focus on the application of the technology for medication management will be included. The titles and abstracts will be reviewed initially for relevance, and then, the full text will be reviewed for in-depth analysis. The results of the search and inclusion process will be presented in a Preferred Reporting Items for Systematic Review and Meta-Analysis extension for Scoping Reviews (PRISMA-ScR) flow diagram. Data will be extracted from the included studies using a data extraction tool developed for this study.

**Results:**

A draft charting table will be developed as a data extraction tool. Results will be presented in a graph, diagram, or table, accompanied by a narrative summary.

**Conclusions:**

This review will identify and synthesize evidence associated with applying technology-based medication management interventions to older people in home care settings and the strategies to overcome those identified challenges. This review will identify knowledge gaps, clarify concepts, and make recommendations for future research on technology-based medication management interventions.

## Introduction

### Overview

Improvement in living conditions and advancement in health sciences have led to people living longer [1]. This has resulted in a rapid increase in the aging population worldwide. With advancing age, people tend to develop problems affecting dexterity, vision, hearing, and cognitive functions, which can significantly affect their ability to take medications or follow instructions, presenting unique challenges that require our understanding and empathy [2]. Furthermore, many older adults are living with multiple health problems (multimorbidity) [1] and are taking multiple medications (polypharmacy) [3]. In addition to health care needs, many older adults have complex social care needs, and they receive care from health care professionals and/or social care support workers in their home settings [4].

The process of medication management involves the identification of a problem, confirmation of a diagnosis, reviewing (starting, stopping, continuing, or changing) medications, and monitoring their effects [5]. Adopting a person-centered and holistic approach is crucial in the process of medication management. This approach not only ensures safe practices but also enhances the health outcomes of older adults [6]. Maidment et al [7] proposed interventions to empower older adults and informal carers to manage the challenges associated with multimorbidity and polypharmacy, which involve identifying older people at risk, maintaining a personalized record of their treatments and health conditions, and sharing it with the individual, informal carer, and practitioners [7].

Managing medicines in home care settings is a complex and challenging task, given the unique home environment and situations that differ from hospital or care home settings [8]. Some older adults self-administer their drugs, whereas others get help from informal caregivers (such as family members, friends, and neighbors) or from social care support workers who may not have the necessary training to manage medications [4]. The roles and responsibilities of individuals involved in providing informal care vary, which may possibly affect communication and increase the risk of medication errors [9].

Problems could potentially occur at any stage of medication use while prescribing, dispensing, storing, administering, or monitoring drugs [10,11]. Some of the challenges associated with medication management in home care settings are non-adherence [12], administration errors, inappropriate storage [13,14], and adverse side effects [15,16]. Additionally, factors including multimorbidity and polypharmacy cause an increased risk of drug-to-drug interactions and drug-to-disease interactions in older adults [3]. These medication-associated problems can affect their health outcomes, causing frailty, falls, frequent hospitalizations, reduced quality of life, and life-threatening events [3]. These challenges lead to increased use of emergency services and hospital beds [17] and can impact health care costs [7].

In recent years, technology-enabled processes such as the use of electronic health records, computerized decision support systems (CDSS), electronic prescriptions, robotic dispensing, electronic medication administration records, and regular telephone follow-up/review have been introduced to overcome problems associated with medication management, offering hope and optimism for the future of geriatric care [11,18]. Additionally, electronic pillboxes, text message reminders, and smartphone applications have been used to enhance medication adherence in older adults [12].

Despite the introduction of these strategies, the problems associated with medication management, such as the incidence of medication error, are high in home care settings compared to primary and secondary care [8,19,20]. Therefore, it is crucial to understand the role of technology-based interventions in managing medications of older adults in home care settings. This will help identify and promote safe medication administration practices. The study will focus on the quality of care, including safety, impact, and accessibility of technology-based medication management interventions [11,21]. The term “impact” refers to “positive or negative, direct or indirect, intended or unintended change produced by an intervention” [22] and the term “accessibility” means availability and ease of use.

A scoping review is an appropriate methodology for this study as they are designed to examine available evidence in the subject area, map key concepts, identify gaps, and develop potential research questions without following strict inclusion criteria [23-25]. Recent reviews on the use of Digital Health Twin (scoping review) [26] and Quality Improvement Intervention (systematic review) [27] in older care settings have demonstrated expected results, that is, improvements in the user implementation of the technologies. A similar review in the setting of older adult care would generate new knowledge for the health and social care system, as well as for older individuals and care providers. The findings of the review, with their broader relevance, have the potential to significantly inform and improve technology-based medication management systems for older adults, thereby meeting current societal challenges.

A preliminary search of the Cochrane Database of Systematic Reviews, Campbell Systematic Reviews, PROSPERO International Prospective Register of Systematic Reviews, and Joanna Briggs Institute (JBI) Evidence Synthesis was conducted. A systematic/scoping review (CRD42023481881-PROSPERO International Prospective Register of Systematic Reviews) is ongoing to explore the impact of nursing roles and responsibilities in optimizing nurse-led digital medication management in older people’s home care. However, this proposed review is different as it will focus on technology-based medication management in older people receiving formal (paid) or informal (family, friends, and neighbors) social care at home and will not include studies involving nursing care. The Cambridge dictionary explains the meaning of social care as “care by public organizations and private companies for people in society who need special help in order to live comfortably, for example, help with washing or eating” [28]. Nursing care in this review refers to care provided by qualified (registered) nurses.

### Aim and Review Questions

The primary objective of this scoping review is to assess and synthesize best available evidence on the use of various technology-based interventions (such as electronic pillboxes, text message reminders, smartphone applications, and electronic medication administration records) in managing medications of older people living in their own home settings with or without the support of formal or informal carers. The quantitative synthesis will identify different types of interventions being used and the qualitative synthesis will focus on understanding the impact of using these interventions on older adults and their carers. The review also aims to explore factors that affect the use of technology-based medication management interventions and the strategies that have been implemented to overcome challenges associated with these interventions. The review questions specifically are: (1) What technology-based interventions are used to support older people living at home and their carers (formal and informal) in managing polypharmacy? (2) What are the impacts of digital health tools (or technology-based interventions) on older people living in home care settings and their carers (formal or informal) in managing medicines? (3) What are the factors (facilitators and barriers) that affect technology-based interventions used for managing medicines for older people living at home? (4) What strategies have been evaluated and implemented in home care settings to address the challenges and improve technology-based medication management interventions?

## Methods

The proposed scoping review will be conducted under the JBI methodology for scoping reviews [29].

### Search Strategy

A database search will retrieve both published and unpublished studies. A three-step search strategy will be followed to find studies for this review (Table 1). The first step would involve conducting an initial limited search of MEDLINE (via PubMed), the Cumulative Index to Nursing and Allied Health Literature (CINAHL), and Scopus to identify articles on the topic. Thereafter, text words in the titles and abstracts of relevant articles, and the index terms used to describe the articles, will be analyzed to develop a complete search strategy. The second step will involve identifying keywords and index terms in each database and/or information source, including Web of Science, EMBASE, IEEE Xplore, and ACM digital library. Finally, the reference list of all included sources of evidence will be screened for additional studies. The EBSCO Open Dissertations database will be used to retrieve unpublished studies, such as dissertations and theses. Reviews, such as systematic, scoping, and narrative reviews, and letters to editors will be excluded. Studies published in all languages will be included in the review.

**Table 1.** Search strategy on databases.

Participant, concept, and context scheme (#)	Search string	Number of articles retrieved on database search using keywords						
		MEDLINE	CINAHL^a^	Scopus	Web of Science	Embase	IEEE Xplore	ACM Digital Library
Medication (#1)	medication or drug* or medicine*	14,838,278	1,391,268	12,482,052	6,913,972	26,186,006	183,753	97,200
Older adults (#2)	aged or elderly or “old* adult*” or geriatric* or “old* people” or “old* person”	6,598,273	1,226,233	7,217,331	5,207,581	6,965,179	112,529	220,136
Home care settings (#3)	“Home care” or “domiciliary care” or “home based care” or “domestic care” or “informal care” or “home care” or “social care”	142,058	119,349	131,091	95,683	140,035	10,117	1,945
Digital technology (#4)	“Digital technology” or “technolog* interventions” or “technolog* solutions” or telehealth or telemedicine or “digital health” or “digital medicine” or ehealth or mhealth	118,974	55,126	237,816	164,600	155,221	733, 312	23,375
Combine (#5)	# 1, #2, #3 AND #4	713	101	369	212	1,273	231	549

a CINAHL: Cumulative Index to Nursing and Allied Health Literature.

### Eligibility Criteria

#### Overview

This scoping review will include the following PCC mnemonics: population, concept, and context. These mnemonics will be used as a guide; therefore, the inclusion criteria of this scoping review will include a detailed description of types of participants, concepts, and context, as well as search strategies, data extraction, charting, analysis, and presentation of the results. The eligibility criteria are listed in Textbox 1.

Textbox 1. **Inclusion and exclusion criteria.**

**Inclusion criteria**

- Conference papers.
- Grey literature.
- Studies involving older individuals (60 years or more) associated with technology-based intervention to manage polypharmacy, regardless of gender, age, ethnicity, socioeconomic status, disorders, or disability.
- Studies that include paid or unpaid carers, whether they are family members or friends.
- Studies that include social care providers involved in older care settings and technology-based intervention to manage polypharmacy.
- Studies that evaluate and discuss the process and application of technology-based intervention to manage polypharmacy involving caregivers, older individuals, or family, friends, or relatives.

**Exclusion criteria**

- Meeting abstracts.
- Review articles.
- Editorial materials.
- Book chapters.
- Studies that do not involve older people living in home care settings.
- Studies that involve older people living in home with support of nursing care
- Studies that do not focus on technology-based interventions in managing polypharmacy or are not directly related to older people living at home with the support of formal or informal social care.
- Studies not in the context of home social care settings.
- Studies where technology-based interventions are not used.

#### Participants

This review will include studies of older people (aged 60 y or more) living at home and managing polypharmacy by themselves or with the assistance of formal (paid social care) or informal (family, friends, and neighbors) care support, irrespective of diversity, including age, gender, race, ethnicity, socio-economic status, and disability. Studies involving older adults receiving home nursing or medical care will not be included. Receiving home nursing or medical care in this review means receiving care from a qualified (registered) health professional, such as a registered nurse or physician.

#### Concept

This review will include studies that evaluate technology-based interventions (digital health tools) to support polypharmacy in older people living at home.

#### Context

Studies that evaluate the effectiveness of medication management in older people in a home care setting will be included. Home care setting in this review means living at own home and receiving social care. Medication management studies involving older people living in nursing homes or care homes or living at home with the support of home nursing care or hospital-at-home care will be excluded from the review.

### Types of Sources

This scoping review will consider both experimental and quasi-experimental study designs, including randomized controlled trials, non-randomized controlled trials, before and after studies and interrupted time-series studies. In addition, analytical observational studies, including prospective and retrospective cohort studies, case-control studies and analytical cross-sectional studies, will be considered for inclusion. This review will also consider descriptive observational study designs, including case series, individual case reports and descriptive cross-sectional studies for inclusion.

Qualitative studies will also be considered, including but not limited to, designs such as phenomenology, grounded theory, ethnography, qualitative description, and action research. Text and opinion papers will also be considered.

### Source of Evidence Selection

Following the search, all identified citations will be collated and exported to Covidence, and duplicates will be removed using this software. In addition to this, during the screening process, duplicates will be removed by manually checking all the retrieved (published and unpublished) articles. A pilot test of the study selection process will be conducted to ensure the efficacy and reliability of the process. During this phase, team members will independently assess up to 25 randomly selected titles and abstracts for alignment with the eligibility criteria. Any discrepancies in the findings, modifications to the eligibility criteria, and additional clarifications or broadening of the document’s scope would be collaboratively reviewed by the entire team. Formal screening would commence on achieving a consensus threshold exceeding 75% as recommended in the JBI scoping review guideline [30].

Following a pilot test, titles and abstracts will then be screened by two or more independent reviewers for assessment against the inclusion criteria for the review. The quality of unpublished articles will be established by checking if ethical principles have been followed. Potentially relevant sources will be retrieved in full, and their citation details will be imported into the JBI System for the Unified Management, Assessment and Review of Information (JBI SUMARI) [30,31]. The full text of selected citations will be assessed in detail against the inclusion criteria by two or more independent reviewers. Reasons for excluding sources of evidence in full text that do not meet the inclusion criteria will be recorded and reported in the scoping review. Any disagreements between the reviewers at each stage of the selection process will be resolved through discussion or with an additional reviewer(s). The results of the search and the study inclusion process will be reported in full in the final scoping review and presented using the Preferred Reporting Items for Systematic Reviews and Meta-Analyses extension for Scoping Reviews (PRISMA-ScR) checklist [32] and flow diagram [33]. Consistency and agreement between reviewers will be evaluated by measuring the inter-rater reliability score using Cohen’s kappa coefficient statistical method [30].

### Data Extraction

Data will be extracted from papers included in the scoping review by two or more independent reviewers using a data extraction tool developed by the reviewers. Translation tools such as Google Translate and Deep L will be used to translate articles published in other (non-English) languages. The data extracted will include specific details about the participants, concept, context, study methods, and key findings relevant to the review question(s).

A draft extraction form will be developed. Some critical information that will be included in the charting table is (but is not limited to): name of the author(s), year of publication, country of origin for the study, aims, study population and sample size, methodology or methods, type of intervention or comparator, duration of the intervention, outcomes measures, and findings. Data on key variables such as technology-based interventions (electronic pillboxes, smartphone applications, text message reminders and electronic medication administration records) used by older adults and their formal/ informal carers, its accessibility (availability of the device, internet connections, and skills to use the device), and its impact including medication adherence, medication errors, and hospitalization will be collected.

Pilot testing of the data extraction form will be conducted on each type of evidence, such as primary research articles, conference papers, and theses. During this stage, each reviewer will complete a data extraction form for a minimum of two to three articles per evidence source type. Thereafter, the reviewers will establish whether it captures the necessary data to answer the review questions. The reviewers will determine if any clarification or modification of the form is required. The draft data extraction tool will be modified and revised as necessary during the process of extracting data from each included evidence source. Modified data extraction forms will be saved using version control and will be shared with all reviewers. Modifications will be detailed in the scoping review. Any disagreements that arise between the reviewers will be resolved through discussion or with an additional reviewer(s). If appropriate, authors of papers will be contacted to request missing or additional data, where required.

## Results

The evidence presented would align with the scoping review objective and questions. Data items will be analyzed by quantifying text and counting the frequency of data extraction items using descriptive statistics, such as percentages /proportions. Frequency counting will be used to determine the number of evidence sources that used a particular technology-based intervention to support older people living at home and their carers (formal and informal) in managing polypharmacy, and to determine the country where the study was conducted [34]. Other relevant information will be extracted by mapping interventions to the Template for Intervention Description and Replication (TIDieR) checklist and by answering the items in the checklist using responses such as “fully reported,” “partially reported,” or “not reported” [35]. Qualitative evidence will be analyzed using a basic qualitative content analysis involving an inductive approach [34].

The data will be presented graphically or in diagrammatic or tabular form. A narrative summary will accompany the tabulated and/or charted results and describe how the results relate to the objective and questions of the review [36].

## Discussion

### Overview

A rapid increase in the aging population is seen globally [37]. This has led to many people living at home with complex social and/or health care needs. The risk of medication-associated problems is high in older people with chronic illness and polypharmacy [9]. It is estimated that approximately 44% to 76% of people with multiple health conditions do not adhere to prescribed medications [38].

Managing medicines in home care settings presents a unique set of challenges, distinct from hospital or care home settings. The complex and dynamic home environment requires innovative solutions for effective medication management. Technology-based interventions have a vast potential to support the health and well-being of older adults [37]. A systematic review of studies on technology-mediated strategies in maintaining medication adherence in older adults identified 19 studies and concluded that strategies, including the use of electronic pillboxes, smartphone applications, and text message reminders, significantly help older adults adhere to complex medication regimes [12].

These advancements hold promise for the future of geriatric care, instilling hope and optimism in the potential of technology-based interventions. Given the high incidence of medication errors in home care settings compared to primary and secondary care, it is crucial to understand the role of technology-based interventions in managing the polypharmacy of older adults in home care settings. This understanding is vital for identifying and promoting safe medication administration practices, underscoring the urgency and importance of this research.

This review will provide information on the availability of different types of technology-based medication management interventions in different countries and the experience of older adults, carers, and health care professionals on their usage. This knowledge will aid in identifying variation in practices and areas for improvement. This study aims to identify the facilitators and barriers to using technology-based medication management interventions in a home care setting by older adults, carers, and health professionals. Understanding the factors that affect its use will help us develop strategies and implement resources to enhance safe practices and promote medication adherence among older adults in home care settings.

### Limitations

It is estimated that by 2050, the majority of older adults will live in low- and middle-income countries [39]. Presently, these countries lack health care and social infrastructure to support older adults, which was evident during the coronavirus disease-19 pandemic [40]. There is a possibility of finding a gap in the literature from low- and middle-income countries on the use of technology-based interventions to support medication management of older adults. The findings of this study should be considered with caution, as there is a possibility of the review being limited to high-income countries. A comprehensive search will be conducted using a standard 3-step method, and gray literature will be included to ensure that all relevant articles are considered for review. The quality, transparency and consistency of the scoping review process will be augmented by using the PRISMA-ScR checklist [32]. Additionally, the recommendations proposed by the Agency for Health Care Research and Quality [41] will be followed to minimize the risk of bias in individual studies.

An inclusive approach will be taken to ensure the scoping review generates generalisable findings [27,42]. However, adequate resources for training and research are required for successful implementation of technology-based medication management interventions [37]. Factors such as a lack of resources and digital inequality in low socio-economic populations [43] and lower- and middle-income countries [44] affect the transferability of findings.

The perspectives of health care professionals, patients, and their carers are significant and integral to the review process. However, they were not involved in the process of developing the protocol, which is a limitation of this scoping review. They will be involved in the discussions to support the interpretation of findings and the dissemination of the review.

### Conclusions

Presently, there are no ongoing systematic reviews or scoping reviews on this topic. This review will identify and synthesize evidence associated with the application of technology-based medication management interventions in older people in home care settings, including availability, experience and factors affecting the use of these interventions. Additionally, this study will identify the strategies employed to overcome the identified challenges in home care settings for older adults. This review will identify knowledge gaps, clarify concepts, and make recommendations for future research on technology-based medication management interventions.

## Supplementary material

10.2196/75200 Checklist 1 PRISMA-ScR checklist.

## Abbreviations

CDSS: Computerized Decision Support System
CINAHL: Cumulative Index to Nursing and Allied Health Literature
JBI: Joanna Briggs Institute
PRISMA-ScR: Preferred Reporting Items for Systematic Reviews and Meta-Analyses extension for Scoping Reviews
PROSPERO: International Prospective Register of Systematic Reviews
